# The effect of electronic health record software design on resident documentation and compliance with evidence-based medicine

**DOI:** 10.1371/journal.pone.0185052

**Published:** 2017-09-21

**Authors:** Yasaira Rodriguez Torres, Jordan Huang, Melanie Mihlstin, Mark S. Juzych, Heidi Kromrei, Frank S. Hwang

**Affiliations:** 1 Department of Ophthalmology, Kresge Eye Institute, Detroit, Michigan, United States of America; 2 School of Medicine, Wayne State University, Detroit, Michigan, United States of America; University of Alabama at Birmingham School of Health Professions, UNITED STATES

## Abstract

This study aimed to determine the role of electronic health record software in resident education by evaluating documentation of 30 elements extracted from the American Academy of Ophthalmology Dry Eye Syndrome Preferred Practice Pattern. The Kresge Eye Institute transitioned to using electronic health record software in June 2013. We evaluated the charts of 331 patients examined in the resident ophthalmology clinic between September 1, 2011, and March 31, 2014, for an initial evaluation for dry eye syndrome. We compared documentation rates for the 30 evidence-based elements between electronic health record chart note templates among the ophthalmology residents. Overall, significant changes in documentation occurred when transitioning to a new version of the electronic health record software with average compliance ranging from 67.4% to 73.6% (p < 0.0005). Electronic Health Record A had high compliance (>90%) in 13 elements while Electronic Health Record B had high compliance (>90%) in 11 elements. The presence of dialog boxes was responsible for significant changes in documentation of adnexa, puncta, proptosis, skin examination, contact lens wear, and smoking exposure. Significant differences in documentation were correlated with electronic health record template design rather than individual resident or residents’ year in training. Our results show that electronic health record template design influences documentation across all resident years. Decreased documentation likely results from “mouse click fatigue” as residents had to access multiple dialog boxes to complete documentation. These findings highlight the importance of EHR template design to improve resident documentation and integration of evidence-based medicine into their clinical notes.

## Introduction

The introduction of electronic health records (EHR) to graduate medical education has the potential to aid residency programs in complying with the competencies required by the Accreditation Council for Graduate Medical Education. The ability of an EHR system to provide access to comprehensive clinical data and to improve daily workflow by decreasing clerical tasks and monitoring residents’ learning experience was key in gaining its acceptance [1–5].

Despite decades of EHR implementation, our knowledge about their impact on residents’ learning outcomes remains limited. Most published articles consist of mixed positive and negative perceptions, anecdotes, and clinical experiences. The impact of EHR on residents’ clinical notes is mixed [6–10]. Previous studies have shown that transitioning to EHR can improve quality of clinical notes, yet its pre-formatted templates, copy–paste function, and auto-filled data have an unknown impact on residents’ clinical reasoning [11]. As the focus on EHR and physician quality reporting continues to expand, it is therefore imperative to evaluate the influence of EHR software design and its upgrades on resident education.

In this study, we aimed to measure the impact of EHR transition and software design on resident education. We used evidence-based elements provided by the American Academy of Ophthalmology (AAO) Preferred Practice Patterns (PPPs) for Dry Eye Syndrome (DES) to measure the quality of the clinical notes. Previous studies examining compliance with PPPs have shown that residents may be at risk for decreased compliance with evidence-based guidelines [12–16]. Monitoring adherence to PPPs provides a guideline for quality patient care and an educational tool for resident education. The goal of our study was to determine if EHR software design played a role in residents’ documentation and compliance with the AAO PPPs for DES.

## Methods

The Institutional Review Board at Wayne State University approved the study protocol. The Kresge Eye Institute (KEI) provided the setting for this study as we underwent a major EHR software upgrade in July of 2013. The EHR database was reviewed for adult patients 18 years old and older with an International Classification of Diseases, Ninth Revision, diagnosis of DES (375.15) examined in the KEI resident clinic between September 1, 2011, and March 31, 2014. Patient charts were excluded if they had a prior diagnosis of DES before September of 2011. One thousand three hundred and seven (1,307) patient charts were identified, and randomization was performed using Excel software (Microsoft, Redmond, WA). A total of 331 patient charts completed by a total of 17 resident physicians who evaluated patients were selected for this study. The KEI ophthalmology residency had seven residents in each year of a three-year ophthalmology residency. Residents were categorized based on postgraduate years (PGY); therefore, first-year residents are PGY2, second-year residents are PGY3, and third-year residents are PGY4. The patient charts selected for the study were evaluated for demographic data and documentation of 30 elements extracted from the AAO PPPs on DES (2011 edition) [12]. These elements were chosen based on their level of evidence and relevance to the diagnosis, treatment, and patient education of DES [12]. The elements were grouped into four sections that comprise the ophthalmological clinical notes: past medical history, physical exam, management, and patient education. The EHR software utilized in this study was NextGen; there was no auto-population or pre-population of elements in the EHR software. We labeled Electronic Health Record A (EHR-A) as the software used before June of 2013 and Electronic Health Record B (EHR-B) as the upgraded software thereafter.

Documentation of elements was collected from all the charts and then categorized as compliant, non-compliant, or not applicable. Information was determined to be not applicable for some cases; for example, “menopausal” only applied to women, and “referral given” only applied if systemic symptoms were present. Data were analyzed using SPSS (IBM, Armonk, NY) statistical analysis software version 21, and Excel version 2013 was used for data tabulation. Statistical methods included utilizing SPSS Generalized Multivariate Analysis of Variance (GMANOVA) and Excel. Average compliance and standard deviation were calculated through Excel analysis of the data and verified with SPSS to ensure accuracy. GMANOVA was used to examine each element as compliant or non-compliant between the major units of analysis, EHR-A and EHR-B. Statistical analysis was also performed for potential confounding variables such as individual resident compliance and resident level of training (i.e., PGY year) between EHR-A and EHR-B. A subset analysis using Unpaired Student T-test was used to evaluate independent factors between these groups, which included patient demographics (age, race, and gender).

## Results

### Descriptive results

A total of 331 patients with a diagnosis of DES in their charts were evaluated by a total of 17 ophthalmology residents, who evaluated patients in both EHR-A and EHR-B. Each resident evaluated an average of 19.47 ± 7.48 study visits. There were 213 charts in the EHR-A group and 118 in the EHR-B group. The mean age of the patients in EHR-A was 49.38 ± 17.09, and that in EHR-B was 49.69 ± 17.13 (p = 0.863). There was no significant difference in gender in this study (p = 0.676) with 228 male (68.88%; 146 in EHR-A; 82 in EHR-B) and 103 female participants (31.12%; 67 in EHR-A; 36 in EHR-B). Table 1 contains a summary of the differences in template design between EHR A and B.

**Table 1:** Differences in documentation methods of different chart note template versions.

Preferred Practice Pattern Element	Documentation Method	
	EHR-A	EHR-B
***Past Medical History***		
Exacerbating conditions	Documentation field available in the Chief Complain/HPI template. No dialog box required.	Documentation field available in the Intake template. Requires opening dialog box under the Reason for Visit section to complete documentation.
Ocular medications and effect on symptoms	Documentation field available in the Histories template. No dialog box required.	Documentation field available in the Intake template. Requires opening dialog box under Medications section to complete documentation.
Ocular surface disease, ocular trauma, and ocular surgical history	Documentation field available in the Histories template. No dialog box required.	Documentation field available in the Histories template. Requires opening dialog box under Past Ocular History to complete documentation.
Contact lenses wear	Documentation field not available in the Examination template. Requires opening dialog box under Special Exams titled CL Tech and CL Examination.	Documentation field available in the Provider template. No dialog box required. Additionally, the Contact Lens Wear field is available in the Prelim Exam template. This method requires opening a dialog box titled Contact Evaluation to complete documentation. Access to Contact evaluation was present both at the top and bottom of the Prelim Exam template.
Smoking exposure	Documentation field available in the Chief Complain/HPI template. No dialog box required.	Documentation field available in the Histories template. Requires opening dialog box under Social History section to complete documentation.
***Physical Exam***		
Skin examination	Documentation field not available in the examination template. Requires opening dialog box in the Plastics section to complete documentation fields.	No specific skin documentation field available in the Prelim Exam and Provider template.
Eyelids and eyelashes	Documentation field available in the Examination template. No dialog box required.	Documentation field available in the Physical Exam section for the Prelim Exam and Provider template. No dialog box required.
Adnexa, puncta, and proptosis	No specific fields present within the Examination template	Documentation field available in the Physical Exam section for the Prelim Exam and Provider template. No dialog box required.

### Resident compliance results

The mean compliance for the 30 elements was 69.62 ± 7.59% (n = 331) for all charts, 68.44 ± 6.42% (n = 146) for PGY2, 70.28 ± 7.48% (n = 63) for PGY3, and 70.69 ± 8.72% (n = 122) for PGY4. The total mean compliance rates between residency years were not statistically significant (p = 0.122). A summary of the results showing the documentation percentage for the 30 elements in each residency year is included in Table 2. Documentation rates were high (>90%) for 12 elements, which included 6 elements of past medical history, 5 of physical exam, and 1 of patient education. There was no statistically significant difference for any of the elements analyzed based on resident year or individual resident. There were no data recorded for menopause and cautioning patients that LASIK may worsen DES. However, none of the patients in the study were noted to be considering LASIK at the time of diagnosis with DES.

**Table 2:** Overall average documentation by resident year.

Preferred Practice Pattern Element	% Documentation				
	All Years (n = 331)	First Year (PGY2) (n = 146)	Second Year (PGY3) (n = 63)	Third Year (PGY4) (n = 122)	*p*
***History***					
Ocular signs and symptoms	96.36	98.62	95.24	94.26	**0.998**
Exacerbating conditions	73.03	79.31	76.19	63.93	**0.674**
Duration of symptoms	80.61	81.38	76.19	81.67	**0.231**
Ocular medications and effect on symptoms	80.36	87.67	71.43	76.23	**0.247**
Ocular surface disease	86.40	91.10	80.95	83.61	**0.909**
Ocular trauma	86.40	91.10	80.95	93.61	**0.909**
Ocular surgical history	86.40	91.10	80.95	83.61	**0.909**
Contact lens wear	26.67	15.18	30.16	38.52	**0.617**
Facial washing (eyelash and eyelid hygiene)	1.52	1.38	1.59	1.64	**0.766**
Systemic medications	92.15	89.04	93.65	95.08	**0.160**
Systemic medical history	96.08	96.58	96.83	95.08	**0.139**
Systemic surgical history	96.07	96.58	96.83	95.08	**0.139**
Allergies	93.05	93.15	93.65	92.62	**0.636**
Menopause	0.0^a^	0.0^a^	0.0^a^	0.0^a^	**N/A**^a^
Smoking exposure	91.54	97.26	85.71	87.70	**0.640**
*Total History Documentation*	74.06	75.73	72.62	72.81	**0.943**
***Physical Exam***					
Best corrected visual acuity	96.97	95.86	96.83	98.36	**0.677**
Skin examination	80.66	93.84	61.90	74.59	**0.688**
Cranial nerve examination	0.0	0.0	0.0	0.0	**N/A**
Eyelids and eyelashes	97.89	99.32	96.83	96.72	**0.587**
Adnexa	31.12	6.85	49.21	50.82	**0.899**
Puncta	31.12	6.85	49.21	50.82	**0.899**
Proptosis	31.12	6.85	49.21	50.82	**0.899**
Conjunctiva	99.70	99.32	100	100	**0.437**
Cornea	99.70	99.32	100	100	**0.437**
Tear film	99.70	99.32	100	100	**0.437**
*Total Physical Exam Documentation*	66.78	60.72	70.32	72.21	**0.943**
***Care Management***					
Address contributing factors	70.10	76.03	74.60	60.66	**0.629**
***Patient Education***					
Counsel on chronic nature	3.32	2.05	15.87	5.74	**0.104**
Instructions on treatment regimen	99.10	98.63	100	99.18	**0.699**
Refer if systemic symptoms are present	66.67 ^b^	66.67^b^	N/A^b^	N/A^b^	**N/A**^b^
Caution that LASIK may worsen symptoms	N/A^c^	N/A^c^	N/A^c^	N/A^c^	**N/A**^c^
*Total Patient Education Documentation*	66.78	60.72	60.32	72.21	**0.277**
***All Elements***					
*Total Documentation*	69.62	68.44	70.28	70.69	**0.946**

^a^Only the 229 female patients included in this study were analyzed for documentation of menopause (100 for PGY-2, 38 for PGY-3, 91 for PGY-4).

^b^Only three of the patients included in this study were analyzed for documentation of referral in the case that systemic symptoms were present (three for PGY-2).

^c^Element was not analyzed as no patients included in this study were considering LASIK.

### EHR-based compliance results

Charts were divided according to EHR-A or EHR-B, showing a significant change in mean compliance from EHR-A (n = 213) to EHR-B (n = 118) with an increase of 67.39 ± 6.20% for EHR-A to 75.63 ± 10.63% for EHR-B (p < 0.0005). A summary of the results showing the documentation percentage for the 30 elements per EHR is presented in Table 3. Overall, EHR-A had high compliance (>90%) in 13 elements while EHR-B had high compliance (>90%) in 11 elements. The transition from EHR-A to EHR-B led to a significant difference in documentation with an increase in documenting contact lens wear from 10.33 ± 30.50% to 56.41 ± 49.80% (p < 0.0005) as well as documenting adnexa, proptosis, and puncta, all increasing from 2.82 ± 16.58% to 82.20 ± 38.41% (p < 0.0005). Conversely, a significant decrease occurred in documentation of smoking exposure from 98.59 ± 11.81% to 78.81 ± 41.04% (p < 0.0005), presence of allergies from 95.31 ± 21.20% to 88.98 ± 31.44% (p = 0.033), and skin examination from 94.37 ± 23.11% to 55.93 ± 49.86% (p < 0.0005). The remaining elements did not show statistically significant changes between EHR.

**Table 3:** Documentation percentage of different chart note template versions.

Preferred Practice Pattern Element	EHR-A (n = 213)	EHR-B (n = 118)	*p*
***History***			
Ocular signs and symptoms	98.12	93.16	**0.331**
Exacerbating conditions	79.34	48.86	**0.135**
Duration of symptoms	80.28	39.24	**0.452**
Ocular medications and effect on symptoms	93.10	75.42	**0.300**
Ocular surface disease	89.67	80.51	**0.171**
Ocular trauma	89.67	80.51	**0.171**
Ocular surgical history	96.24	80.51	**0.171**
Contact lens wear	10.33	56.41	**<0.0005**
Facial washing (eyelash and eyelid hygiene)	1.88	0.85	**0.110**
Systemic medications	90.61	94.92	**0.999**
Systemic medical history	96.24	95.76	**0.799**
Systemic surgical history	96.24	95.76	**0.799**
Allergies	95.31	88.98	**0.033**
Menopause	0.0^a^	0.0^a^	**N/A**^a^
Smoking exposure	98.59	78.81	**<0.0005**
*Total History Documentation*	74.84	72.65	**0.310**
***Physical Exam***			
Best corrected visual acuity	95.75	99.15	**0.293**
Skin examination	94.37	55.93	**<0.0005**
Cranial nerve examination	0	0	**N/A**
Eyelids and eyelashes	99.53	94.92	**0.075**
Adnexa	2.82	82.20	**<0.0005**
Puncta	2.82	82.20	**<0.0005**
Proptosis	2.82	82.20	**<0.0005**
Conjunctiva	99.53	100.00	**0.824**
Cornea	99.53	100.00	**0.824**
Tear film	99.53	100.00	**0.824**
*Total Physical Exam Documentation*	59.65	79.66	**<0.0005**
***Care Management***			
Address contributing factors	70.42	69.49	**0.090**
***Patient Education***			
Counsel on chronic nature	1.88	5.93	**0.364**
Instructions on treatment regimen	99.06	99.15	**0.792**
Refer if systemic symptoms are present	66.67^b^	0.0^b^	**N/A**^b^
Caution that LASIK may worsen symptoms	N/A^c^	N/A^c^	**N/A**^c^
*Total Patient Education Documentation*	50.55	52.54	**0.564**
***All Elements***			
*Total Documentation*	67.42	73.57	**<0.0005**

^a^Only 103 female patients included in this study were analyzed for documentation of menopause (67 for the EHR-A, 36 for EHR-B).

^b^Only three patients included in this study had documentation of referral in the case that systemic symptoms were present.

^c^Element was not analyzed as no patients included in this study were considering LASIK.

Additionally, charts were divided according to the residents’ years in training and compared between EHR chart note templates. A summary of the results showing differences in documentation is presented in S1 Table. When these changes were evaluated based on EHR template design, most remained statistically significant. The results show that significant changes in documentation were related not to the resident year in training but, rather, to EHR template design.

## Discussion

Our study is unique as we measured the effects of EHR software design on resident documentation and compliance with evidence-based medicine. The results showed an increase in overall documentation from 67.42% in EHR-A to 73.57% in EHR-B (p < .001). One of the most noticeable changes with the upgrade from EHR-A to EHR-B was an increase in point-and-click EHR interfaces and dialog boxes. The addition of dialog boxes decreased the number of elements with high compliance (>90%). Overall, EHR-A had high compliance (>90%) in 13 elements while EHR-B had high compliance (>90%) in 11 elements. We postulate these changes were related to template design and the addition of dialog boxes. For example, both EHR-A and B chart note templates included fields for documentation of smoking exposure. Yet, after the software upgrade, EHR-B required the residents to open a separate dialog box under the History section to document smoking exposure. The presence of dialog boxes was responsible for significant changes in documentation of adnexa, puncta, proptosis, skin findings, contact lens use, and smoking exposure.

These findings highlight the importance of EHR software design and its influence on physician documentation. In our study, decreased documentation likely resulted from “mouse click fatigue” as residents had to access multiple dialog boxes. This phenomenon affected all residents regardless of the year in training. Conversely, documentation remained stable for elements in which the EHR template fields were readily available in both EHR-A and B. These findings are comparable to a study by Sanders et al. that reported a worsening in documentation time at their ophthalmology ambulatory clinic and operating room after EHR implementation due to the point-and-click EHR interfaces [17]. This phenomenon is not isolated to ophthalmology as a recent survey of physicians found generalized frustration with EHR on-screen boxes leading to modifications in EHR template design [18]. It is important to note that our observed mouse-clicking fatigue is very similar to alert fatigue, a phenomenon in which clinically insignificant reminders lead to a paradoxical increase in patient safety hazards. Therefore, EHR software design must be carefully evaluated for these phenomena as they can decrease physician compliance with standards of care [19].

Within residency training programs, monitoring compliance with guidelines ensures that residents provide quality patient care early in their careers and integrate evidence-based medicine into their practice [15, 20, 21]. Increasing compliance and minimizing EHR barriers are important as they can lead to fewer patient complications and possible reduction in overall cost of medical care [20, 22]. Academic institutions and program directors should be aware of barriers to appropriately plan for these major technological transitions and minimize adverse effects [23, 24]. Our study provided a measure of residents’ knowledge of DES and use of patient education and identified EHR barriers to delivering quality documentation. These results provide objective evidence that can aid in improving the quality of graduate medical education, which can subsequently result in direct improvement of patient care.

This study had several limitations. Our study was based on a single ophthalmology resident clinic at one academic institution; thus, the results may not be generalizable to other clinic settings. EHR software versions compared in the study are among many commercially available EHR programs, and our results in compliance may not be generalizable to other EHR software. This is a retrospective chart review; hence, data were limited to what was documented in the EHR. Lastly, the number of charts documented by individual residents was low, limiting the ability to detect significant differences in individual resident documentation. Similar to other published ophthalmology resident compliance studies, we are unable to correlate our findings with clinical outcomes. Therefore, future trials are needed to study the correlation between residents’ adherence to evidence-based guidelines and improved patient care.

Our study has several strengths. This study included a sample of 331 charts that spanned over four years and included 17 residents at different years of training. All the elements measured in our study are supported by a body of evidence and were extracted directly from the AAO PPPs on DES (2011 edition). This is the first study to demonstrate that EHR template design can significantly affect the quality of clinical notes documented by residents.

## Conclusions

The content and quality of the EHR chart note template play important roles in guiding documentation. EHR design factors can be responsible for the success or failure of adherence to evidence-based guidelines [25, 26], Therefore, as the focus on EHR and physician quality reporting continues to expand, it becomes imperative to evaluate the influence of EHR software design and its upgrades on resident education. Our study shows that EHR software design does have a significant impact on the quality of the residents’ clinical note.

Before this study and other PPP-related studies performed in our clinic, there was limited emphasis on practice and implementation of PPP guidelines in our clinic. Awareness of the impact of EHR design and continued emphasis on PPPs have led to EHR modifications, the first being a link within the EHR template to AAO PPPs guidelines and references. Additionally, an educational workshop on PPPs was implemented last year, allowing residents to self-evaluate their compliance with PPPs and providing the opportunity for practice-based learning. Currently, our clinical transformation team is working on modifying documentation templates to include key elements from PPPs. Future studies will determine the impact of these interventions on compliance and how compliance relates to improvement of patient care. Additional studies will include coordination with EHR companies to create templates for graduate medical education that aid in developing resident competencies and achievement of milestones.

## Supporting information

S1 Table Resident year vs. EHR version. (DOCX)
