# Supplementary material for: The effect of electronic health record software design on resident documentation and compliance with evidence-based medicine
Source: PLoS One. 2017 Sep 21;12(9):e0185052. doi: 10.1371/journal.pone.0185052 (PMC5608474; doi:10.1371/journal.pone.0185052)
Supplement: S1 Table — (DOCX) [file pone.0185052.s001.docx]

**S1 Table. Resident year vs. EHR version.**

| **Preferred Practice Pattern Element** | **Documentation (%)** | | | | | | | | |
| --- | --- | --- | --- | --- | --- | --- | --- | --- | --- |
|  | **First Year (PGY2)** | | | **Second Year (PGY3)** | | | **Third Year (PGY4)** | | |
|  | **EHR-A (n = 139)** | **EHR-B (n = 50)** | ***p*** | **EHR-A (n = 31)** | **EHR-B (n = 32)** | ***p*** | **EHR-A (n = 43)** | **EHR-B (n = 80)** | ***p*** |
| ***History*** | | | | | | | | | |
| Ocular signs and symptoms | 98.56 | 100.00 | *0.768* | 96.77 | 93.75 | *0.231* | 97.67 | 92.41 | *0.231* |
| Exacerbating conditions | 79.86 | 66.67 | *0.445* | 83.87 | 68.75 | *0.082* | 74.42 | 58.23 | *0.082* |
| Duration of symptoms | 80.58 | 100.00 | *0.243* | 74.19 | 78.13 | *0.775* | *83.72* | 81.01 | *0.442* |
| Ocular medications and effect on symptoms | 87.05 | 100.00 | *0.348* | 74.19 | 68.75 | *0.925* | *76.74* | 75.95 | *0.925* |
| Ocular surface disease | 90.65 | 100.00 | *0.434* | 87.10 | 75.00 | *0.287* | *88.37* | 81.01 | *0.287* |
| Ocular trauma | 90.65 | 100.00 | *0.434* | 87.10 | 75.00 | *0.287* | *88.37* | 81.01 | *0.287* |
| Ocular surgical history | 90.65 | 100.00 | *0.434* | 87.10 | 75.00 | *0.287* | *88.37* | 81.01 | *0.287* |
| Contact lens wear | 13.67 | 50.00 | *0.016* | 0.00 | 59.38 | *<0.0005* | *6.98* | 55.70 | *<0.0005* |
| Facial washing (eyelash and eyelid hygiene) | 1.43 | 0.00 | *0.769* | 3.22 | 0.00 | *0.672* | *2.33* | 1.27 | *0.672* |
| Systemic medications | 93.55 | 100.00 | *0.380* | *95.35* | 93.75 | *0.566* | 90.61 | 94.94 | *0.899* |
| Systemic medical history | 96.40 | 100.00 | *0.638* | 93.55 | 93.75 | *0.302* | *97.67* | 94.94 | *0.302* |
| Systemic surgical history | 96.40 | 100.00 | *0.638* | 93.55 | 100.00 | *0.302* | *97.67* | 93.67 | *0.302* |
| Allergies | 93.53 | 85.71 | *0.342* | 100.00 | 87.50 | *0.095* | *97.67* | 89.87 | *0.095* |
| Menopause | 0.00 | 0.00 | *n/a* | 0.00 | 0.00 | *n/a* | *0.00* | 0.00 | *n/a* |
| Smoking exposure | 98.56 | 71.43 | *0.00* | 96.77 | 75 | *0.002* | *100* | 81.01 | *0.002* |
| *Total History Documentation* | 68.44 | 81.70 | *0.440* | 70.28 | 72.07 | *0.376* | 70.69 | 72.09 | *0.376* |
| ***Physical Exam*** | | | | | | | | | |
| Best corrected visual acuity | 95.86 | 100.00 | *0.605* | 96.83 | 100.00 | *0.668* | 98.36 | 98.73 | *0.6688* |
| Skin examination | 93.84 | 57.14 | *0.002* | 64.90 | 37.50 | *<0.0005* | 74.59 | 63.29 | *<0.0005* |
| Cranial nerve examination | 0.00 | 0.00 | *n/a* | 0.00 | 0.00 | *n/a* | *0.00* | 0.00 | *n/a* |
| Eyelids and eyelashes | 99.32 | 100.00 | *0.836* | 96.83 | 93.75 | *0.142* | 96.72 | 94.94 | *0.142* |
| Adnexa | 6.85 | 71.43 | *<0.0005* | 49.21 | 93.75 | *<0.0005* | 50.82 | 78.48 | *<0.0005* |
| Puncta | 6.85 | 71.43 | *<0.0005* | 49.21 | 93.75 | *<0.0005* | 50.82 | 78.48 | *<0.0005* |
| Proptosis | 6.85 | 71.43 | *<0.0005* | 49.21 | 93.75 | *<0.0005* | 50.82 | 78.48 | *<0.0005* |
| Conjunctiva | 99.32 | 100.00 | *0.836* | 100.00 | 100.00 | *n/a* | 100.00 | 100.00 | *n/a* |
| Cornea | 99.32 | 100.00 | *0.836* | 100.00 | 100.00 | *n/a* | 100.00 | 100.00 | *n/a* |
| Tear film | 99.32 | 100.00 | *0.836* | 100.00 | 100.00 | *n/a* | 100.00 | 100.00 | *n/a* |
| *Total Physical Exam Documentation* | 60.72 | 77.14 | *<0.0005* | 70.32 | 81.25 | *<0.0005* | 72.21 | 79.24 | *<0.0005* |
| ***Care Management*** | | | | | | | | | |
| Address contributing factors | 76.03 | 57.14 | *0.538* | 74.60 | 78.13 | *0.048* | 60.66 | 67.09 | *0.048* |
| ***Patient Education*** | | | | | | | | | |
| Counsel on chronic nature | 2.05 | 0.00 | *0.717* | 1.59 | 3.13 | *0.260* | 5.74 | 7.59 | *0.260* |
| Instructions on treatment regimen | 98.63 | 100.00 | *0.768* | 100.00 | 100.00 | *0.495* | 99.18 | 98.74 | *0.495* |
| Refer if systemic symptoms are present | 66.67 | 0.0 | *N/A* | 0.0 | 0.0 | *N/A* | 0.0 | 0.0 | *N/A* |
| *Total Patient Education Documentation* | 50.46 | 50.00 | *0.905* | 50.79 | 51.56 | *0.442* | 52.46 | 53.16 | *0.442* |
| ***All Elements*** | | | | | | | | | |
| *Total Documentation* | 68.45 | 76.31 | *0.005* | 70.28 | 74.10 | *<0.0005* | 70.69 | 73.12 | *<0.0005* |
